# Supplementary material for: Spatiotemporal Analysis for COVID-19 Delta Variant Using GIS-Based Air Parameter and Spatial Modeling
Source: Int J Environ Res Public Health. 2022 Jan 30;19(3):1614. doi: 10.3390/ijerph19031614 (PMC8835317; doi:10.3390/ijerph19031614)
Supplement: Supplementary file 1 [file ijerph-19-01614-s001.zip › ijerph-1506908-supplementary.pdf]

International Journal of
Environmental Research and Public Health

MDPI

Supplementary Materials:

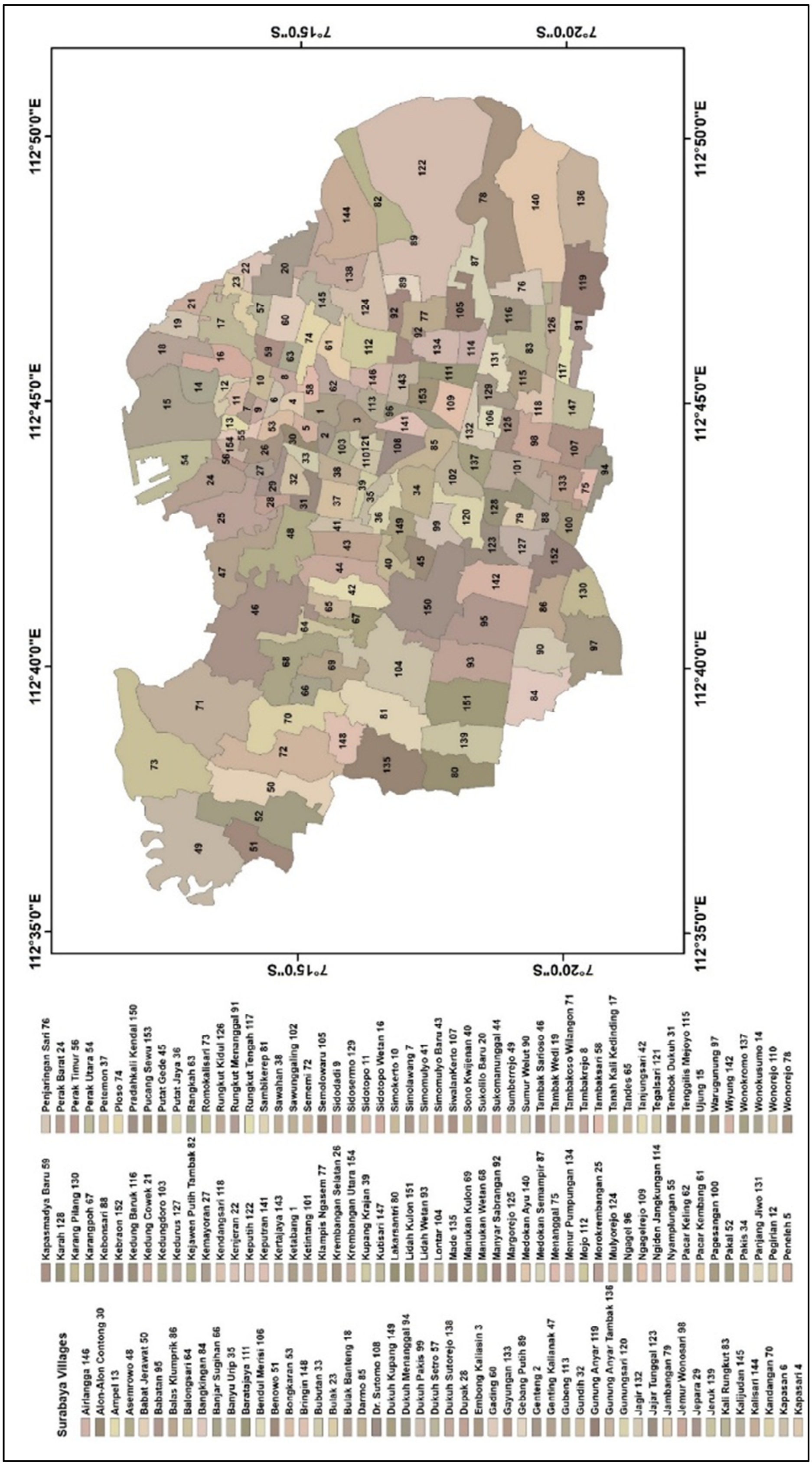

Figure S1. Village's Administration of Surabaya as study area.

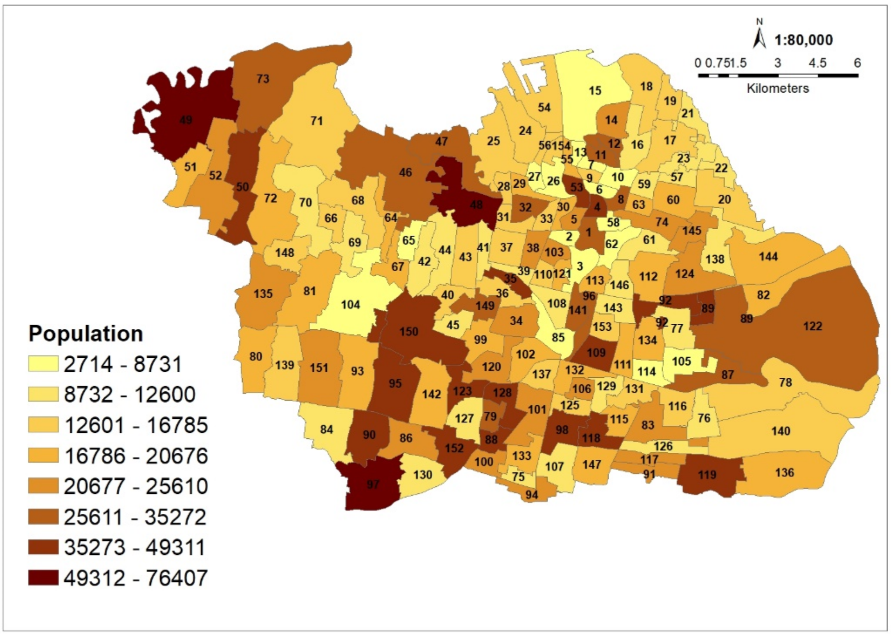

(**a**)

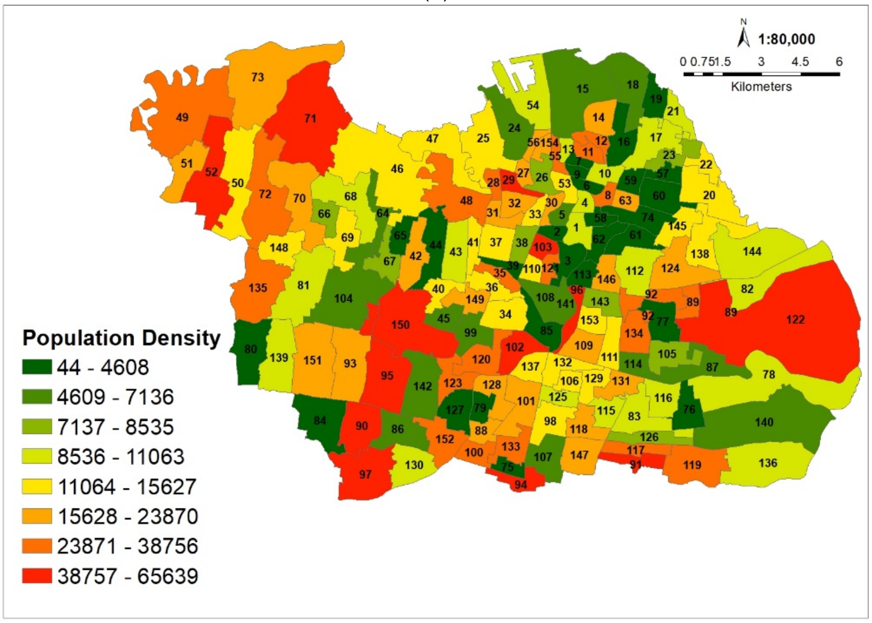

(**b**)

Figure S2. Population Distribution of Surabaya on 2020 (**a**) Population Distribution of Each Village (**b**) Distribution of Population Density (population/km2) of Each Village.

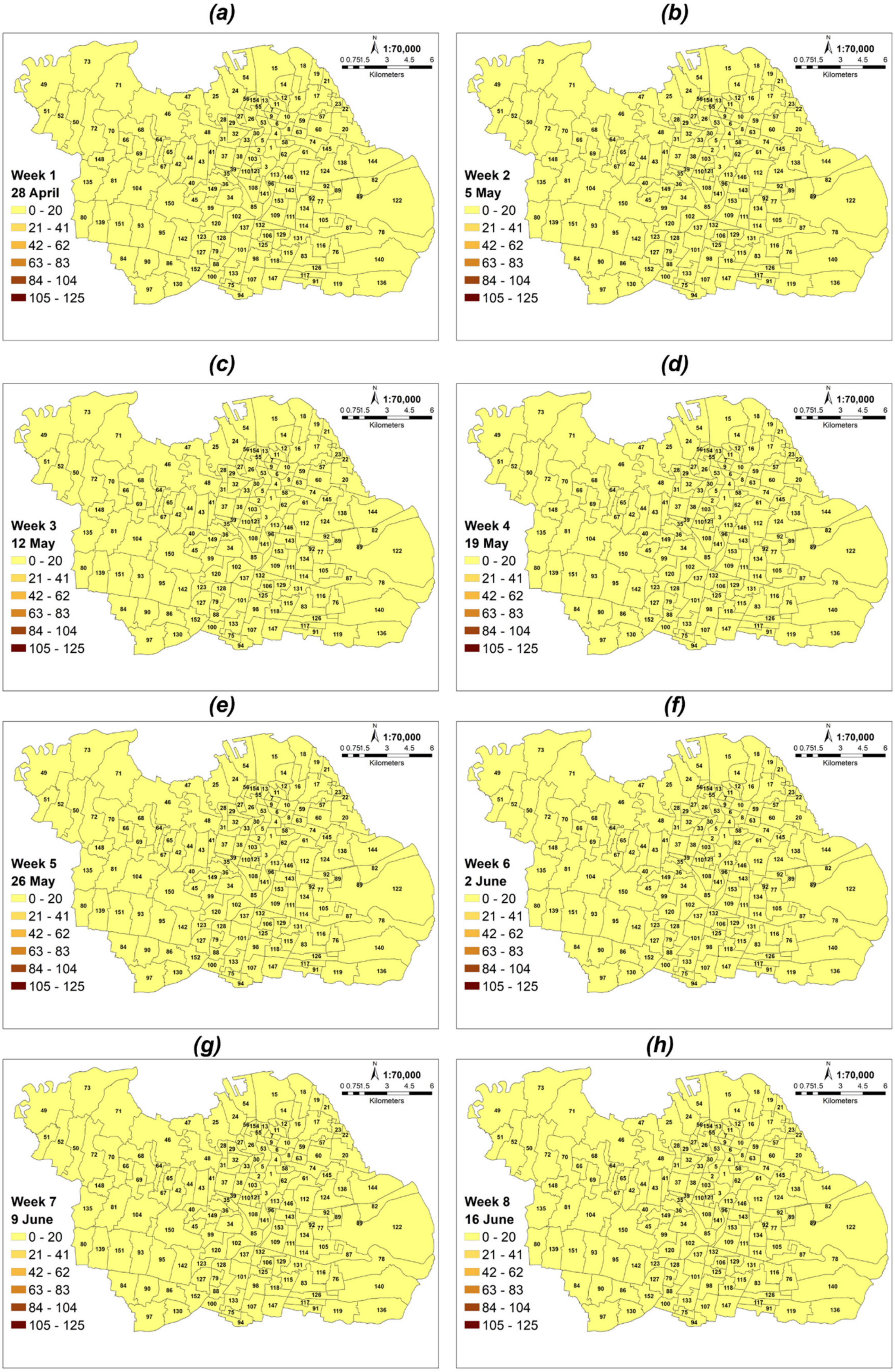
(a)
1:70,000
0 0.75 1.5 3 4.5 6
Kilometers
Week 1
28 April
0 - 20
21 - 41
42 - 62
63 - 83
84 - 104
105 - 125
(b)
Week 2
5 May
(c)
Week 3
12 May
(d)
Week 4
19 May
(e)
Week 5
26 May
(f)
Week 6
2 June
(g)
Week 7
9 June
(h)
Week 8
16 June

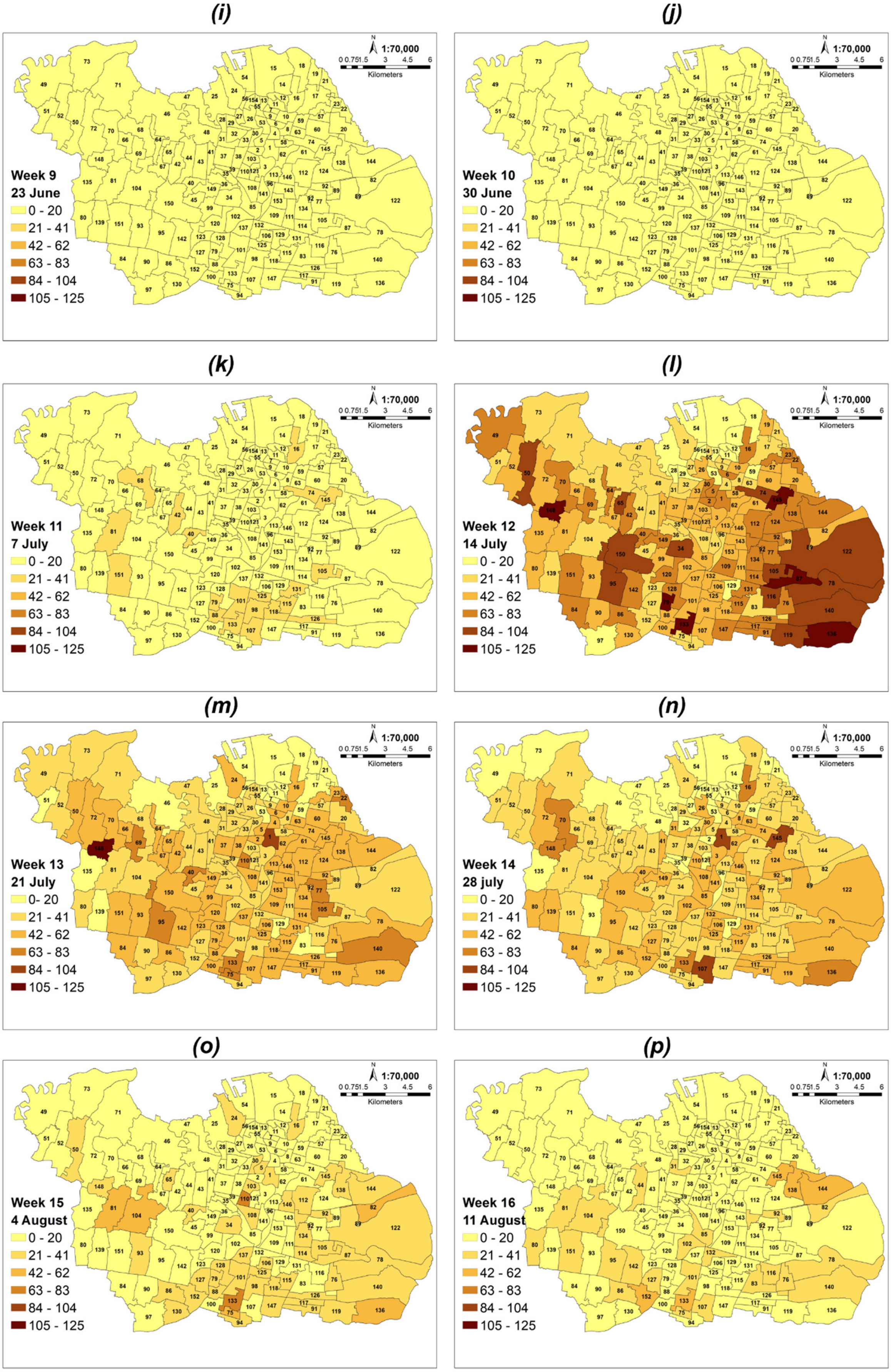
(i)
1:70,000
0 0.75 1.5 3 4.5 6
Kilometers
Week 9
23 June
0 - 20
21 - 41
42 - 62
63 - 83
84 - 104
105 - 125
(j)
1:70,000
0 0.75 1.5 3 4.5 6
Kilometers
Week 10
30 June
0 - 20
21 - 41
42 - 62
63 - 83
84 - 104
105 - 125
(k)
1:70,000
0 0.75 1.5 3 4.5 6
Kilometers
Week 11
7 July
0 - 20
21 - 41
42 - 62
63 - 83
84 - 104
105 - 125
(l)
1:70,000
0 0.75 1.5 3 4.5 6
Kilometers
Week 12
14 July
0 - 20
21 - 41
42 - 62
63 - 83
84 - 104
105 - 125
(m)
1:70,000
0 0.75 1.5 3 4.5 6
Kilometers
Week 13
21 July
0- 20
21 - 41
42 - 62
63 - 83
84 - 104
105 - 125
(n)
1:70,000
0 0.75 1.5 3 4.5 6
Kilometers
Week 14
28 july
0 - 20
21 - 41
42 - 62
63 - 83
84 - 104
105 - 125
(o)
1:70,000
0 0.75 1.5 3 4.5 6
Kilometers
Week 15
4 August
0 - 20
21 - 41
42 - 62
63 - 83
84 - 104
105 - 125
(p)
1:70,000
0 0.75 1.5 3 4.5 6
Kilometers
Week 16
11 August
0 - 20
21 - 41
42 - 62
63 - 83
84 - 104
105 - 125

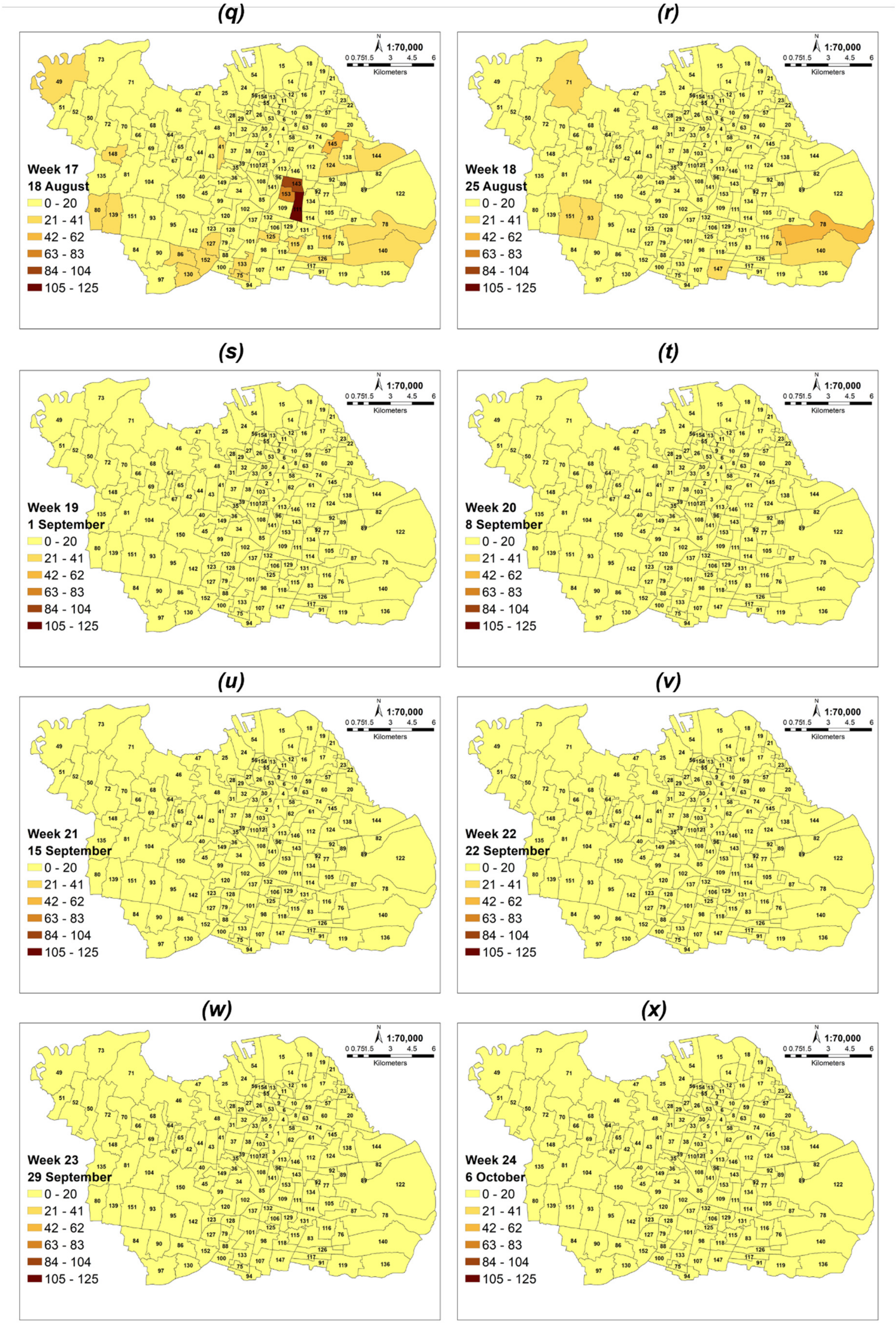
(q)
Week 17
18 August
(r)
Week 18
25 August
(s)
Week 19
1 September
(t)
Week 20
8 September
(u)
Week 21
15 September
(v)
Week 22
22 September
(w)
Week 23
29 September
(x)
Week 24
6 October
0 - 20
21 - 41
42 - 62
63 - 83
84 - 104
105 - 125
1:70,000
0 0.75 1.5 3 4.5 6
Kilometers
N

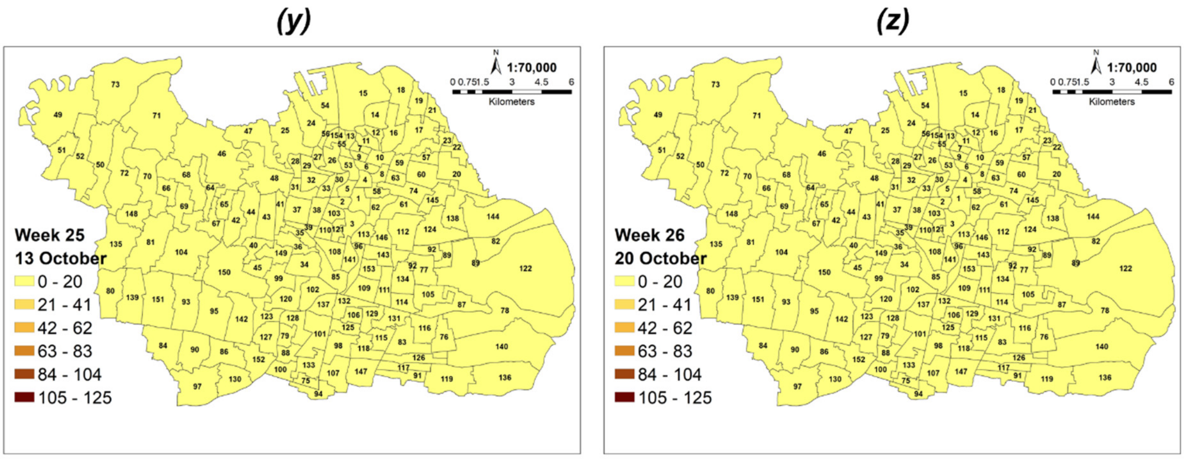

Figure S3. The distribution of confirmed COVID−19 cases per 100,000 populations every week in Surabaya. (**a**) 1st Week (28 April 2021 – 4 May 2021); (**b**) 2nd Week (5 May 2021 – 11 May 2021); (**c**) 3rd Week (12 May 2021 – 19 May 2021); (**d**) 4th Week (20 May 2021 – 25 May 2021); (**e**) 5th Week (26 May 2021 – 1 June 2021); (**f**) 6th Week (2 June 2021 – 8 June 2021); (**g**) 7th Week (9 June 2021 – 15 June 2021); (**h**) 8th Week (16 June 2021 – 22 June 2021); (**i**) 9th Week (23 June 2021 – 29 June 2021); (**j**) 10th Week (30 June 2021 – 6 July 2021); (**k**) 11th Week (7 July 2021 – 13 July 2021); (**l**) 12th Week (14 July 2021 – 20 July 2021); (**m**) Week 13 (21 July 2021 – 27 July 2021); (**n**) Week 14 (28 July 2021 – 3 August 2021); (**o**) 15th Week (4 August 2021 – 10 August 2021); (**p**) 16th Week (11 August 2021 – 17 August 2021); (**q**) 17th Week (18 August 2021 – 24 August 2021); (**r**) 18th Week (25 August 2021 – 31 August 2021); (**s**) 19th Week (1 September 2021 – 7 September 2021); (**t**) 20th Week (8 September 2021 – 14 September 2021); (**u**) 21st Week (15 September 2021 – 21 September 2021); (**v**) 22nd Week (22 September 2021 – 28 September 2021); (**w**) 23rd Week (29 September 2021 – 5 October 2021); (**x**) 24th Week (4 October 2021 – 12 October 2021); (**y**) 25th Week (13 October 2021 – 19 October 2021); (**z**) 26th Week (20 October 2021 – 26 October 2021).

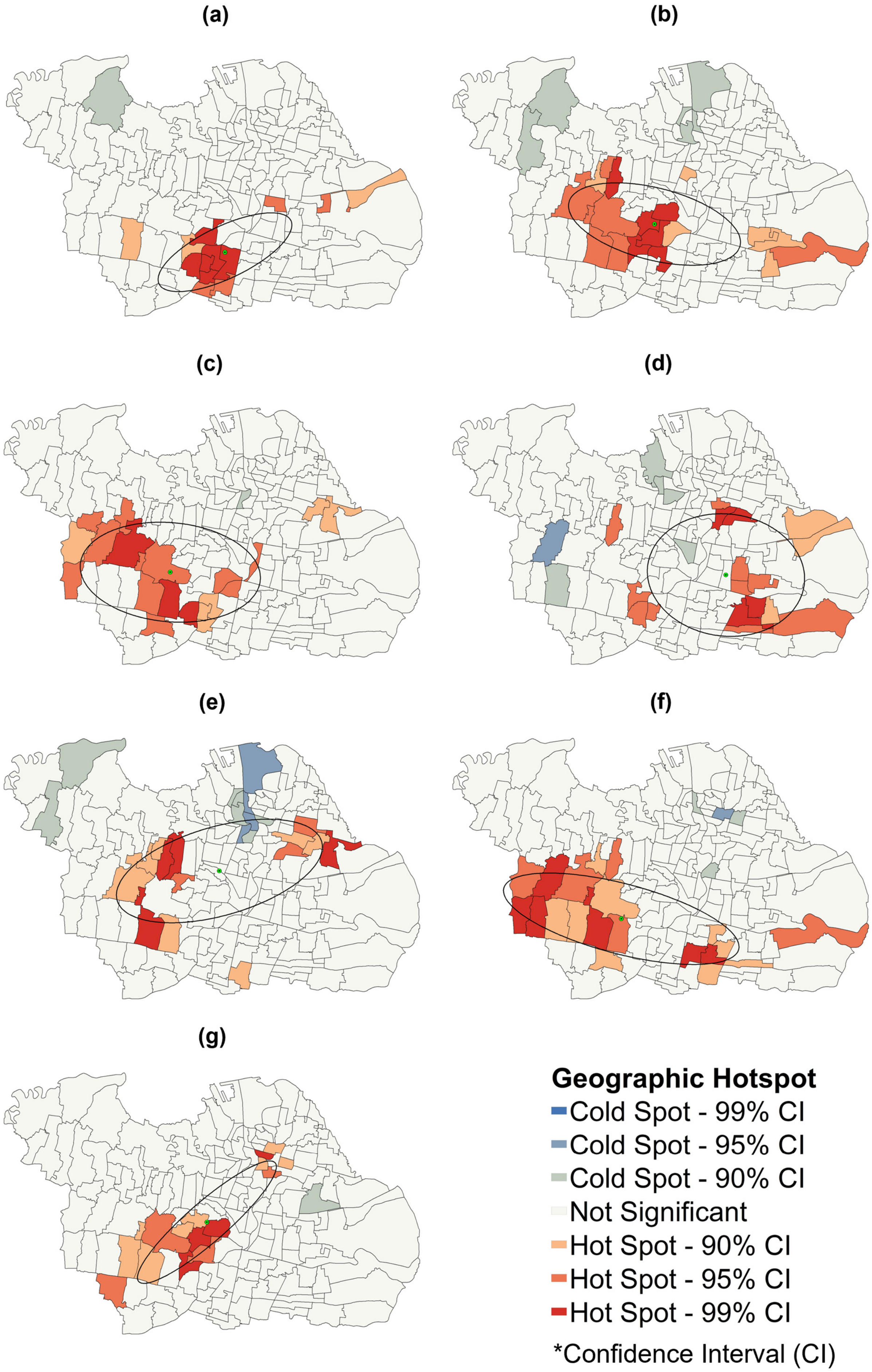
(a)
(b)
(c)
(d)
(e)
(f)
(g)
Geographic Hotspot
Cold Spot - 99% CI
Cold Spot - 95% CI
Cold Spot - 90% CI
Not Significant
Hot Spot - 90% CI
Hot Spot - 95% CI
Hot Spot - 99% CI
*Confidence Interval (CI)

[1]

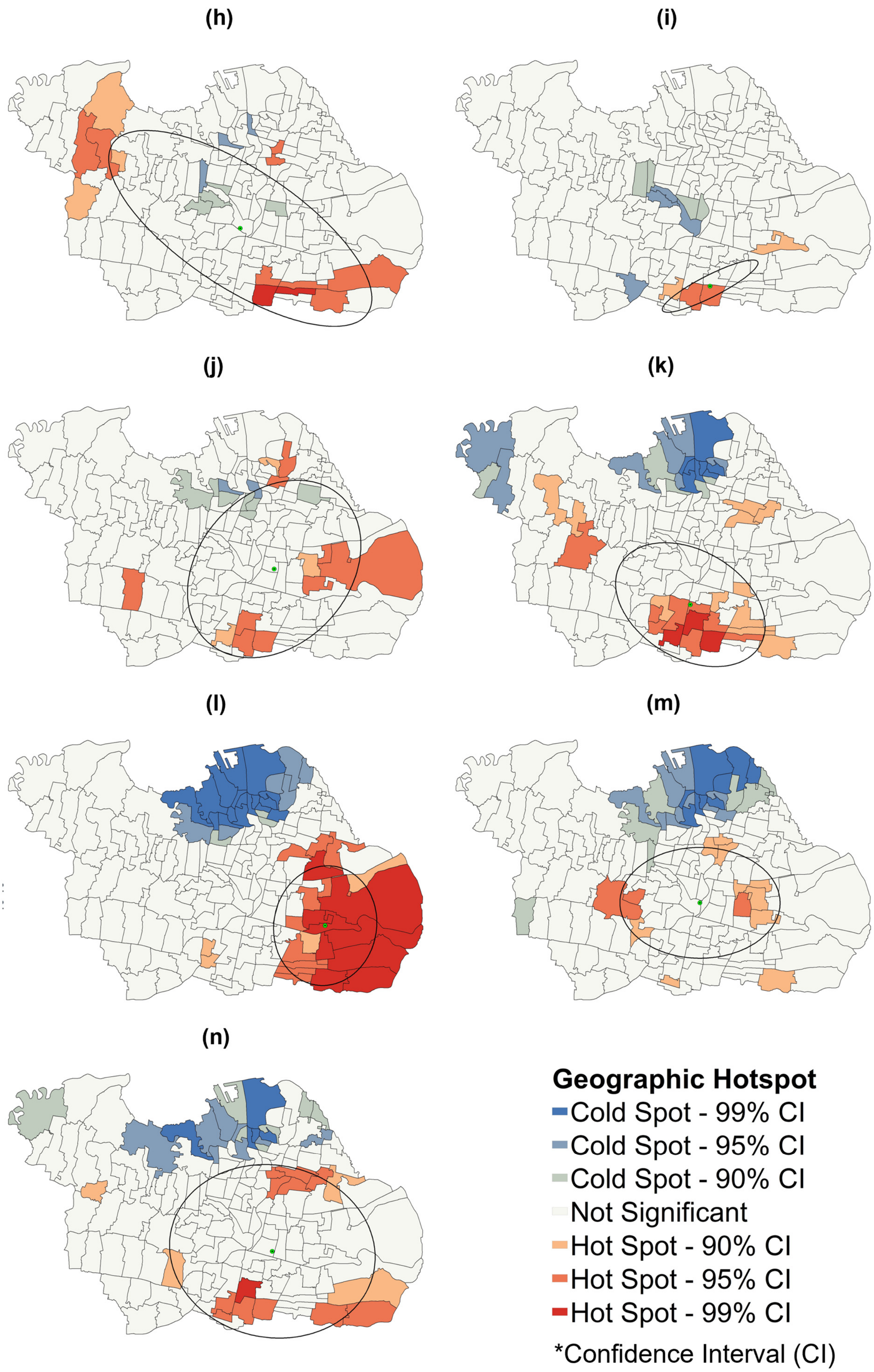
(h)
(i)
(j)
(k)
(l)
(m)
(n)
Geographic Hotspot
Cold Spot - 99% CI
Cold Spot - 95% CI
Cold Spot - 90% CI
Not Significant
Hot Spot - 90% CI
Hot Spot - 95% CI
Hot Spot - 99% CI
*Confidence Interval (CI)

[2]

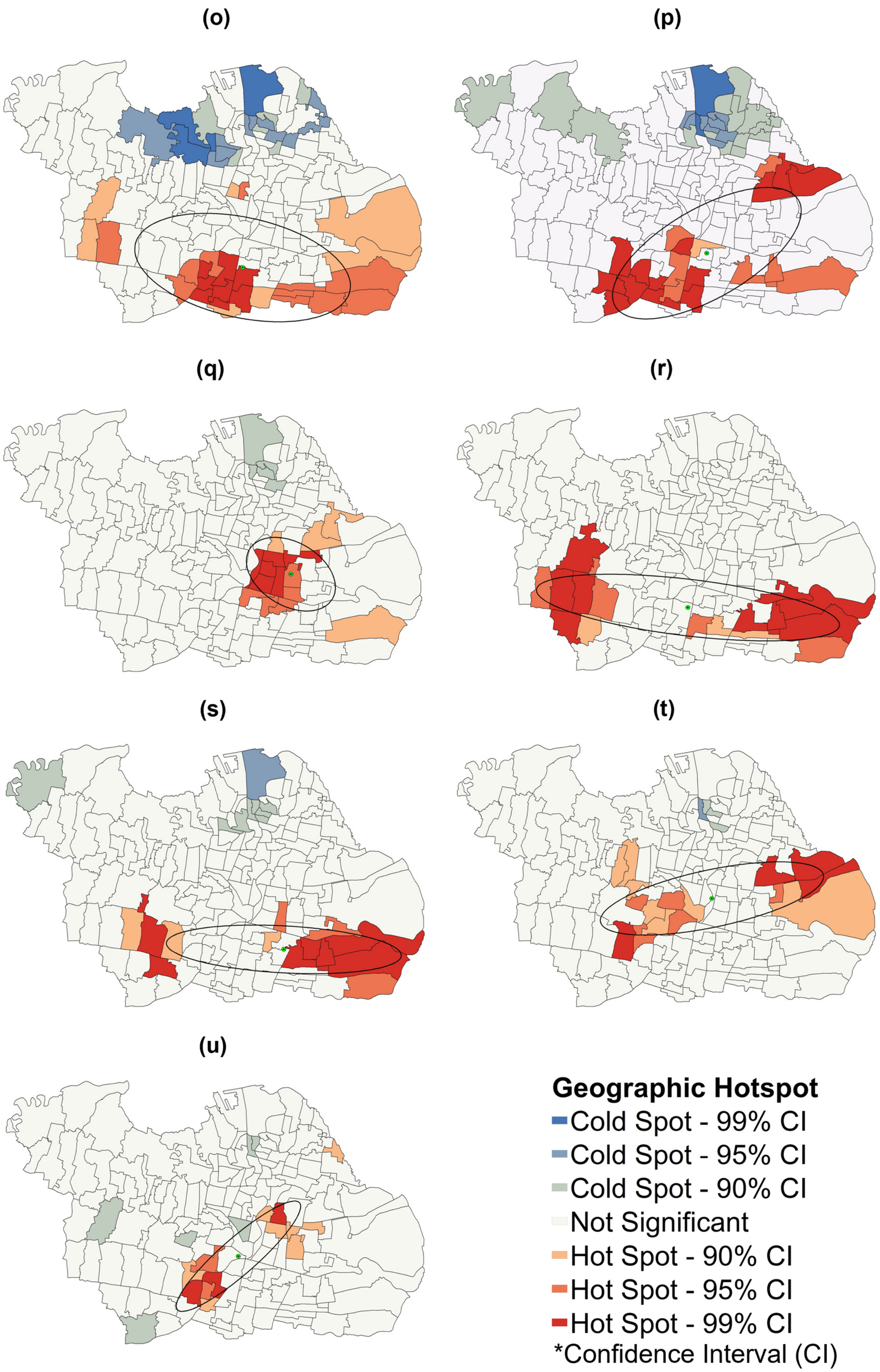
(o)
(p)
(q)
(r)
(s)
(t)
(u)
Geographic Hotspot
Cold Spot - 99% CI
Cold Spot - 95% CI
Cold Spot - 90% CI
Not Significant
Hot Spot - 90% CI
Hot Spot - 95% CI
Hot Spot - 99% CI
*Confidence Interval (CI)

[3]

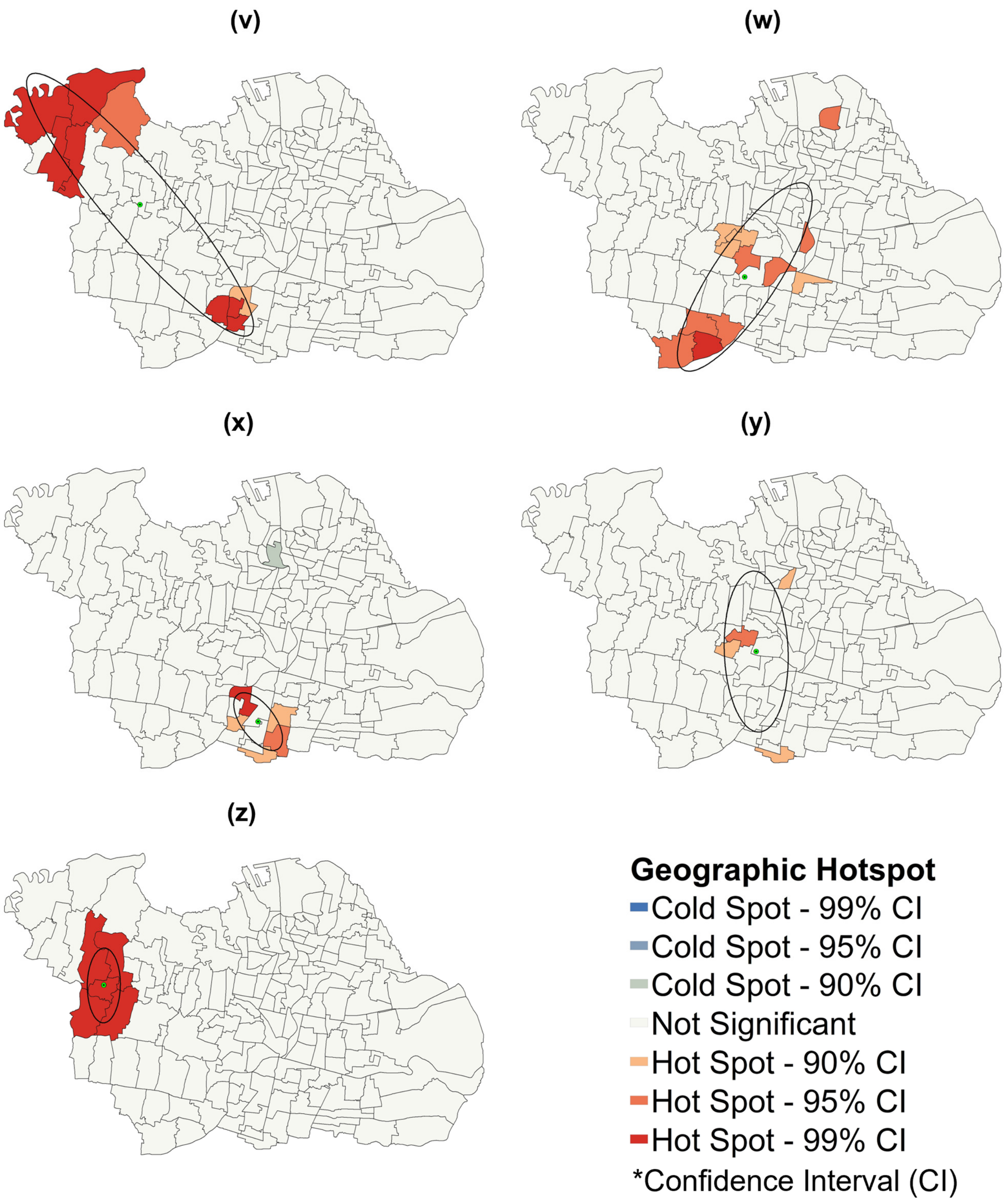

Figure S4. The clustering map of Covid−19 incidence along with its orientation and shift directions. (**a**) 1st Week (28 April 2021 – 4 May 2021); (**b**) 2nd Week (5 May 2021 – 11 May 2021); (**c**) 3rd Week (12 May 2021 – 19 May 2021); (**d**) 4th Week (20 May 2021 – 25 May 2021); (**e**) 5th Week (26 May 2021 – 1 June 2021); (**f**) 6th Week (2 June 2021 – 8 June 2021); (**g**) 7th Week (9 June 2021 – 15 June 2021); (**h**) 8th Week (16 June 2021 – 22 June 2021); (**i**) 9th Week (23 June 2021 – 29 June 2021); (**j**) 10th Week (30 June 2021 – 6 July 2021); (**k**) 11th Week (7 July 2021 – 13 July 2021); (**l**) 12th Week (14 July 2021 – 20 July 2021); (**m**) Week 13 (21 July 2021 – 27 July 2021); (**n**) Week 14 (28 July 2021 – 3 August 2021); (**o**) 15th Week (4 August 2021 – 10 August 2021); (**p**) 16th Week (11 August 2021 – 17 August 2021); (**q**) 17th Week (18 August 2021 – 24 August 2021); (**r**) 18th Week (25 August 2021 – 31 August 2021); (**s**) 19th Week (1 September 2021 – 7 September 2021); (**t**) 20th Week (8 September 2021 – 14 September 2021);

(**u**) 21st Week (15 September 2021 – 21 September 2021); (**v**) 22nd Week (22 September 2021 – 28 September 2021); (**w**) 23rd Week (29 September 2021 – 5 October 2021); (**x**) 24th Week (4 October 2021 – 12 October 2021); (**y**) 25th Week (13 October 2021 – 19 October 2021); (**z**) 26th Week (20 October 2021 – 26 October 2021).

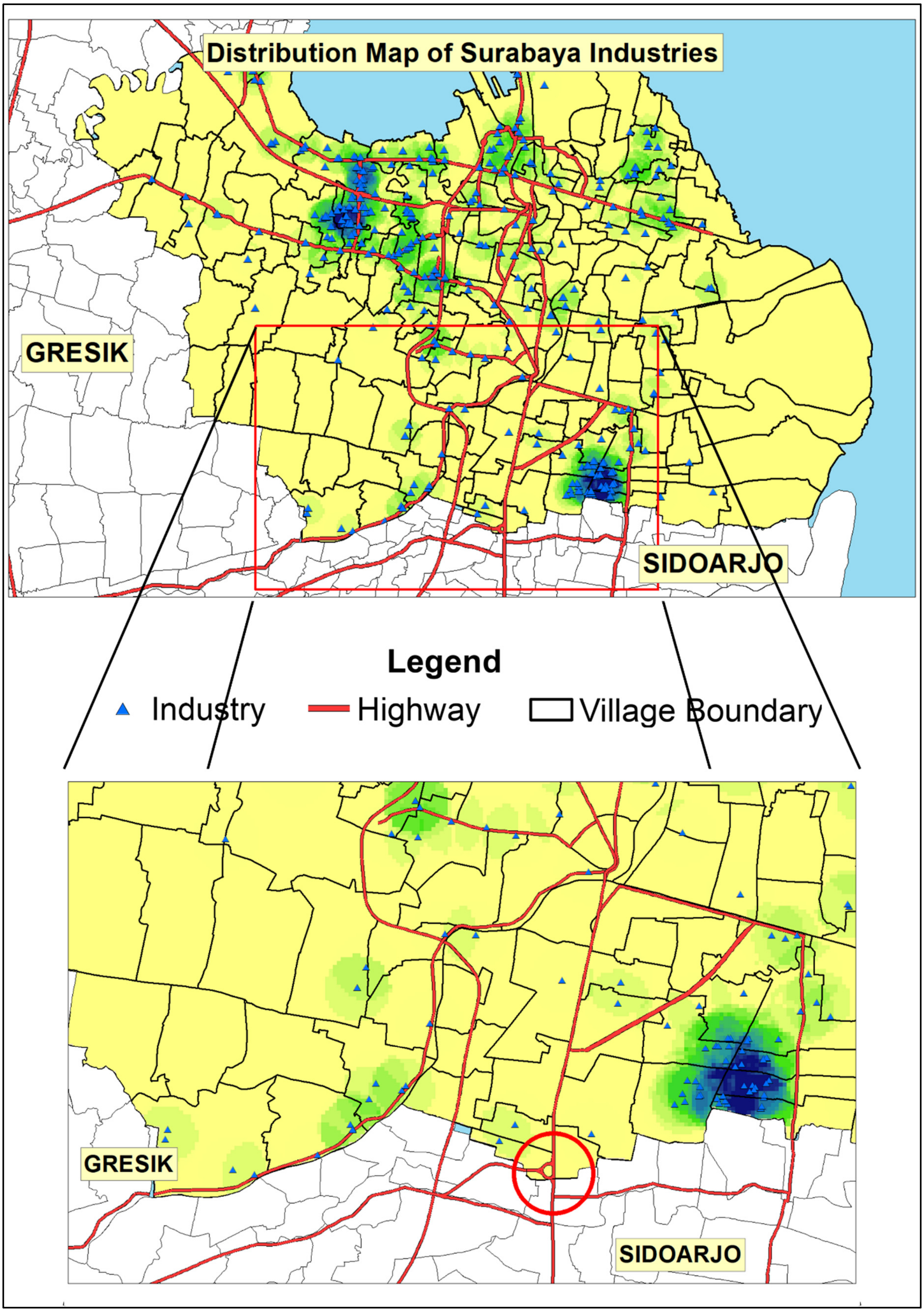

Figure S5. Distribution map of Surabaya Industries. The red circle (bottom) is called the Gerbangkertosusilo area. It is the entrance area to the Surabaya and the surrounding area, includes Gresik–Bangkalan–Mojokerto–Surabaya–Sidoarjo–Lamongan.

Table S1. Location of Weighted Mean Center (WMC).

Week	Date	Coordinat X (m)	Coordinat Y (m)	WMC (Village)
1	28 April 2021 – 4 May 2021	690491.505	9191371.709	Ketintang
2	5 May 2021 – 11 May 2021	689363.660	9193374.052	Dukuh Pakis
3	12 May 2021 – 19 May 2021	686970.804	9193297.146	Pradahkali Kendal
4	20 May 2021 – 25 May 2021	694193.433	9193105.752	Baratajaya
5	26 May 2021 – 1 June 2021	690289.795	9195714.877	Banyu Urip
6	2 June 2021 – 8 June 2021	687118.472	9192483.670	Wiyung
7	9 June 2021 – 15 June 2021	689446.500	9194591.563	Dukuh Kupang
8	16 June 2021 – 22 June 2021	691448.149	9193282.331	Darmo
9	23 June 2021 – 29 June 2021	692959.379	9189365.370	Kutisari
10	30 June 2021 – 6 July 2021	693748.499	9193659.268	Pucang Sewu
11	7 July 2021 – 13 July 2021	691619.805	9191239.394	Margorejo
12	14 July 2021 – 20 July 2021	697205.301	9192097.089	Medokan Semampir
13	21 July 2021 – 27 July 2021	692271.624	9193645.441	Darmo
14	28 July 2021 – 3 August 2021	693622.804	9192651.546	Ngagelrejo
15	4 August 2021 – 10 August 2021	691793.009	9190756.498	Jemur Wonoasri
16	11 August 2021 – 17 August 2021	692674.419	9191722.292	Bendul Merisi
17	18 August 2021 – 24 August 2021	695039.162	9193606.229	Menur Pumpungan
18	25 August 2021 – 31 August 2021	691228.659	9191270.241	Ketintang
19	1 September 2021 – 7 September 2021	694389.574	9190909.745	Tenggilis Mejoyo
20	8 September 2021 – 14 September 2021	692841.732	9194333.037	Keputran
21	15 September 2021 – 21 September 2021	691338.223	9192912.292	Sawunggaling
22	22 September 2021 – 28 September 2021	683675.753	9196653.623	Manukan Kulon

23	29 September 2021 – 5 October 2021	688798.646	9192411.546	Gunungsari
24	4 October 2021 – 12 October 2021	690597.087	9189792.971	Ketintang
25	13 October 2021 – 19 October 2021	689467.776	9193904.644	Pakis
26	20 October 2021 – 26 October 2021	681552.383	9196934.499	Bringin
